# Retraction: Hypoxia Preconditioned Mesenchymal Stem Cells Prevent Cardiac Fibroblast Activation and Collagen Production via Leptin

**DOI:** 10.1371/journal.pone.0279676

**Published:** 2022-12-21

**Authors:** The PLOS ONE Editors

Following the publication of this article and Correction [1, 2], the corresponding author (XH) requested correction of additional errors in Figures 1 and 7. Following editorial assessment, additional issues were identified. Specifically,

- Several partially overlapping panels were identified among files described as raw images underlying replicate samples for Figures 2C and 4C.
- For quantification of fluorescence intensity in Figures 2D and 4D, the corresponding author stated that 6–9 images randomly captured from 3 samples were measured for each group.
- The author reported that the β-actin panel in Figure 1F is incorrect.
- The author reported that the β-actin panels in Figures 4A and 7A are identical, and that Figure 7A is incorrect.

The corresponding author stated that all the raw data underlying the results in this article remain available, and they made the data accessible via Dryad [3].

In relation to the concerns with the files described as raw images underlying Figures 2C and 4C, the corresponding author stated that the experiments were performed at the same time, and the CFs, H-CFs and H-CFs-H-MSCs^WT^ groups are the same in the two figures. They acknowledged that errors occurred in file storage and provided alternative sets of files described as raw images underlying Figures 2C and 4C. The concerns regarding partially overlapping panels persisted in the alternative sets of files. The corresponding author acknowledged that this overlap potentially occurred because multiple fields were randomly captured from individual samples.

The editors remain concerned about the reliability of these data given the errors in file storage and the inclusion of multiple images of the same sample as separate replicates for analysis of fluorescence intensity.

The corresponding author provided updated versions of Figures 1F and 7A in which the β-actin panels have been replaced. The editors consider these issues resolved.

In light of the concerns affecting multiple figures, the *PLOS ONE* Editors retract this article.

WZ, JW, and XH did not agree with the retraction and stand by the article’s findings. PC, RW, ZJ, YX, HaC, ZZ, HuC, LZ, and HY either did not respond directly or could not be reached.

## References

[1] Chen P, Wu R, Zhu W, Jiang Z, Xu Y, Chen H, et al. (2014) Hypoxia Preconditioned Mesenchymal Stem Cells Prevent Cardiac Fibroblast Activation and Collagen Production via Leptin. PLoS ONE 9(8): e103587. doi: 10.1371/journal.pone.0103587 25116394 PMC4130526

[2] Chen P, Wu R, Zhu W, Jiang Z, Xu Y, Chen H, et al. (2015) Correction: Hypoxia Preconditioned Mesenchymal Stem Cells Prevent Cardiac Fibroblast Activation and Collagen Production via Leptin. PLoS ONE 10(12): e0143983. doi: 10.1371/journal.pone.0143983 26633300 PMC4669181

[3] Hu Xinyang (2022) Hypoxia preconditioned mesenchymal stem cells prevent cardiac fibroblast activation and collagen production via leptin. Dryad, Dataset, doi: 10.5061/dryad.sf7m0cg9d PMC4130526 25116394

